# HZ08 suppresses RelB-activated MnSOD expression and enhances Radiosensitivity of prostate Cancer cells

**DOI:** 10.1186/s13046-018-0849-5

**Published:** 2018-07-27

**Authors:** Yanyan Zhang, Zhi Xu, Jiaji Ding, Chunli Tan, Weizi Hu, Yunman Li, Wenlong Huang, Yong Xu

**Affiliations:** 1 Jiangsu Cancer Hospital & Jiangsu Institute of Cancer Research, & The Affiliated Cancer Hospital of Nanjing Medical University, 42 Baiziting, Nanjing, 210009 People’s Republic of China; 2 Jiangsu Key Lab of Cancer Biomarkers, Prevention and Treatment, Nanjing Medical University, Nanjing, 211166 People’s Republic of China; 3 State Key Laboratory of Natural Medicines, Department of Physiology, China Pharmaceutical University, Nanjing, 210009 People’s Republic of China; 4 Center of Drug Discovery, China Pharmaceutical University, Nanjing, 210009 People’s Republic of China

**Keywords:** RelB, MnSOD, HZ08, Radioresistance, Prostate cancer

## Abstract

**Background:**

The development of radioresistance is one of main causes for therapeutic failure of prostate cancer (PCa). The present study aims to investigate the function and the related mechanism by which HZ08 sensitizes radiotherapeutic efficiency to treat aggressive PCa cells.

**Methods:**

PCa cells were pretreated with HZ08 (6,7-dimethoxy-1-(3,4-dimethoxy) benzyl-2-(N-n-octyl-N′-cyano) guanyl-1,2,3,4-tetrahydroisoquinoline) and followed by ionizing radiation (IR) treatment. Cytotoxicity in the treated cells was analyzed to assess the radiosensitization capacity of HZ08 by flow cytometry, MTT and colony survival assays. The cellular levels of reactive oxygen species (ROS) and oxygen consumption rates (OCR) were measured using specific ROS detection probes and a Seahorse XF96 Analyzer, respectively. RelB binding to the NF-κB intronic enhancer region of the human *SOD2* gene was determined using a ChIP assay. The levels of phosphorylation of PI3K, Akt and IKKα were quantified and further confirmed using a PI3K inhibitor. Finally, the synergistic effect of HZ08 on radiosensitization of PCa cells was validated using a mouse xenograft tumor model.

**Results:**

HZ08 enhanced radiosensitivity of PCa cells through increasing ROS and declining mitochondrial respiration due to suppression of mitochondrial antioxidant enzyme MnSOD. Mechanistically, HZ08 appeared to inhibit PI3K/Akt/IKKα signaling axis, resulting in transcriptional repression of MnSOD expression by preventing RelB nuclear translocation.

**Conclusions:**

HZ08 can serve as a useful radiosensitizing agent to improve radiotherapy for treating aggressive PCa cells with high level of constitutive RelB. The present study suggests a promising approach for enhancing radiotherapeutic efficiency to treat advanced PCa by inhibiting antioxidant defense function.

**Electronic supplementary material:**

The online version of this article (10.1186/s13046-018-0849-5) contains supplementary material, which is available to authorized users.

## Background

PCa is the second most common malignancy and a major leading cause of cancer death among men worldwide [1]. Although the 5-year survival rates of PCa steadily increase in the United States, the mortality of PCa has been increasing every year globally, particularly in East Asia [2, 3]. Radiotherapy is well recognized to be one of the most popular treatment options for localized PCa [4]. However, a significant portion of men were diagnosed with advanced stages of PCa that are resistant to the conventional radiotherapy and chemotherapy. Although these patients are treated with the improved radiotherapy, PCa still can relapse after definitive radiotherapy, indicating that intrinsic or acquired radioresistance has occurred during radiotherapy [5, 6]. Since the limitation in radiotherapy for patients with advanced PCa, the therapeutic strategies need to be improved to enhance the radiosensitivity of PCa.

In the past decade, several studies had been conducted to identify radiosensitizing agents that were able to sensitize PCa cells to radiation. However, only three such trials had been reported with the eliminated effects, including two targeted agents and one natural compound. Subsequently, 14 additional trials have recently been completed or are undergoing to treat patients with high-risk PCa by combination of radiotherapy with the selected radiosensitizing agents. Unfortunately, most of the clinic trials have failed to present the promising results due to either low efficacy or unexpected side-effects [7]. Thus, it is a severe challenge to discover clinical useful radiosensitizing agents for the improvement of traditional radiotherapy to treat advanced PCa.

DNA double-strand breaks are considered to be a major consequence of radiotherapy, which can be caused directly by irradiation energy or indirectly through radiation-induced ROS produced from water dissociation. ROS include superoxide radicals (O_2_^•ˉ^), hydroxyl radicals (^•^OH), hydrogen peroxide (H_2_O_2_), as well as thereby generating other downstream oxidative products so on, which participate in DNA damage when the function of the cellular antioxidant system was declined [8, 9]. Notably, approximately two-thirds of radiation-mediated DNA damage is caused by the indirect effects of accumulated ROS [10]. As a primary antioxidant enzyme located in mitochondria, MnSOD plays a key role in protection of cells against ROS by elimination of excessive superoxide radicals [11]. Virtually, it has been proved that adaptive activation of MnSOD is essential for cell survival by detoxification of radiation-induced ROS [12]. Therefore, we argue that the current therapeutic strategies for advanced PCa presumably need to be improved by administration of intracellular redox homeostasis.

Nuclear factor-κb (NF-κB) activation has been implicated in radioresistance of cancers [13]. NF-κB serves as a transcription factor ubiquitously in distribution and regulates a series of gene expression. Five NF-κB family members have been discovered to exist as homodimers or heterodimers: NF-κB1 (p50/p105), NF-κB2 (p52/p100), RelA (p65), RelB, and c-Rel. NF-κB can be activated through either RelA:p50-based classical (canonical) or RelB:p52-based alternative (non-canonical) pathway by a multitude of stimulants including IR [14]. Although most studies have focused on the classical pathway [15–17], we and others found that RelB is uniquely expressed at the high levels in advanced PCa and the levels of nuclear RelB are particularly associated with the patients Gleason scores [18, 19]. Additionally, as a typical NF-κB regulated protein, MnSOD is adaptively stimulated by IR-induced ROS production partially through RelB-mediated transcriptional activation, and selectively preventing RelB nuclear translocation resulted in enhancing radiosensitivity of PCa cells [20] .

HZ08 has been designed and synthesized in our laboratory. Since tetrahydroisoquinoline shows reversal function of drug resistance in cancer treatment, we have tested more than 100 derivatives to identify clinical useful compounds as multidrug resistance modulators [21]. HZ08 displays a novel reverse effect on drug resistance by modulating P-glycoprotein [22–25]. Thus, the present study aims to test whether HZ08 sensitizes radiotherapeutic efficiency to treat aggressive PCa cells. As anticipated, the results indicated that HZ08 is able to enhance radiosensitivity of PCa cells by repression of RelB-regulated MnSOD expression. Mechanistically, prevention of RelB nuclear translocation by inhibition of PI3K/Akt/IKKα phosphorylation is involved in HZ08-mediated radiosensitization. The finding of this study suggests that HZ08 may serve as a useful radiosensitizing agent for treating advanced PCa.

## Methods

### Cell culture and treatments

Human PCa cell lines PC-3 and DU-145 purchased from the American Type Culture Collection (ATCC, USA) were grown and maintained in the recommended medium, supplemented with 10% fetal bovine serum, 100 U/ml penicillin and 100 μg/ml streptomycin. RelB has been permanently silenced by stably transfecting a RelB small interfering RNA (siRNA) construct into PC-3 cells as previously described [26]. A MnSOD expression construct was transfected into PC-3 and DU-145 cells to diminish HZ08-mediated cytotoxicity. To enhance radiotherapeutic efficiency, the cells were pretreated with HZ08 prior to IR treatment using a X-ray machine (Rad Source RS2000 X-ray, USA). HZ08 and PI3K inhibitor LY294002 (Sellectchem, China) were dissolved in DMSO and prepared the storage concentration of 100 mM; and further diluted with culture media to obtain the required concentrations. The final concentration of DMSO in the culture media was less than 0.1% (*v*/v).

### Cell survival analysis

MTT and colony survival assays were performed to quantify cytotoxicity after PCa cells were treated with HZ08, IR or combination. For MTT assay, 5 × 10^3^ cells were cultured in 96-well plates overnight. The cells were pretreated with HZ08 at concentrations of 0 to100 μM for 24 h and then followed by IR treatment at 0–6 Gy. To inhibit PI3K activation, the cells were pretreated with LY294002 at the concentration of 25 μM for 24 h prior to IR treatments. The cells were further cultured for 48 h, and MTT assay was conducted using a microplate reader (Thermo Multiskan, USA) at 570 nm. For colony survival assay, 100–1000 cells were plated in 6-well plates and cultured for 24 h before HZ08 and IR treatment at relating doses corresponding to MTT assay. The treated cells were culture for 7–21 days allowing colony formation. The colonies were stained with 1% crystal violet and counted as previously described [27]. The survival curves were plotted by normalizing with the effect of HZ08 alone.

### Flow cytometry

Flow cytometry analysis was used to quantify apoptotic cells after HZ08 and IR treatment. 2 × 10^5^ cells were seeded in 6-well plates overnight and then treated with HZ08 and IR. One day after treatment, the cells were collected and washed three times with cold PBS. The cells were then stained with 5 μM annexin V-FITC and 2.5 μM PI (Dojindo Molecular Technologies, Japan) in a binding buffer for 15 min at room temperature and dark condition. Apoptotic cells were quantified using a BD FACSCalibur flow cytometer (BD Sciences, USA) and the percentage of apoptotic cells was calculated by number of proapoptotic and apoptotic cells divided by the amount of total cells.

### ROS quantification

A dichlorofluorescein (DCF) assay (Sigma, USA) was used to quantify the levels of intracellular ROS after HZ08 and IR treatment according to the manufacture’s procedure. Briefly, the cells were labeled with DCF-DA to control the testing cell numbers, and then the cells were stained with H_2_DCF-DA, a ROS susceptible form to quantify ROS. The levels of intracellular ROS were assessed by normalizing H_2_DCF-DA images with DCF-DA images to optimize cell number, dry uptake and ester cleavage. The fluorescent images of DCF were quantified using a microplate reader (BioTek synergy 2, USA) at excitation (495 nm) and emission (520 nm). To quantify superoxide anions induced by HZ08, the cells were incubated with 5 μM MitoSOX reagent (Invitrogen, USA) for 10 min at 37 °C. After washing with PBS, the fluorescent images were quantified at excitation (510 nm) and emission (580 nm). To determine the production of superoxide anion, 50 μg SOD (Beyotime Biotechnology, China) or heat-inactivated SOD was added to treat the cells for 1 h prior to HZ08 treatment.

### Measurement of MnSOD activity

Protein extracts from the treated cells and tumor tissues were subjected to measure MnSOD activity using a SOD Assay Kit with WST-8 (Beyotime Biotechnology) according to the manufacture’s instruction. Briefly, the extracts were centrifuged at 12000 rpm at 4 °C for 10 min to remove cell debris and the supernatants were added to a WST-8/Enzyme working buffer containing a Cu/ZnSOD inhibitor at 37 °C for 30 min. The OD values were measured at 450 nm using a microplate reader. The activity of MnSOD was calculated according to the formula provided by the manufacture.

### Quantification of oxygen consumption

To analyze mitochondrial respiration, a Seahorse XF96 Analyzer (Seahorse Biosciences, USA) was used to measure OCR in the treated cells. The cells were transferred into a 96 well XF96 plate at a density of 5 × 10^3^ cells/well and incubated overnight. Cartridge plates for metabolic stress injections were hydrated for 24 h at 37 °C without CO_2_ in calibrant solution. One hour prior to the assay, the medium in the XF96 plate was replaced by Seahorse Assay Media and the assay was performed according to the manufacture’s procedure. OCR was normalized by total cellular protein concentrations.

### Western blots

Cells or tumor tissues were harvested to prepare cellular and nuclear proteins. Total proteins were extracted using a RIPA lysis buffer containing PMSF, and nuclear proteins were extracted using a nuclear and cytoplasmic protein extraction kit (Beyotime Biotechnology) according to the manufacture’s instruction. The extracted proteins (50–100 μg) were separated on SDS-PAGE gels and then transferred to PVDF membranes. The membranes were subsequently incubated overnight at 4 °C with the primary antibodies purchased from Cell Signaling Technology, USA, including RelA, #4764; RelB, #10544; MnSOD, #13141; PI3K, #4249; p-PI3K, #4228; Akt, #9272; p-Akt, #9271; β-actin, #4970 and PCNA, #13110, with an exception of IKKα (ab32041) and p-IKKα (ab17943) purchased from abcam, UK. Thereafter, the membranes were washed three times with TBST buffer and incubated at room temperature for 2 h with HRP-conjugated secondary antibody (Santa Cruze Biotechnology, USA). The immunoblotting was visualized using enhanced chemiluminescence detection system (Bio-Rad ChemiDoc XRS, USA). The intensities of blots were analyzed by Quantity One software and protein expression was normalized by loading controls such as β-actin and PCNA.

### RNA isolation and quantitative reverse transcription polymerase chain reaction (qRT-PCR)

Total RNA was isolated from the treated cells by Tirzol extraction and then converted to cDNA using a PrimeScript RT reagent kit (Takara Biomedical Technology Co., Ltd., Japan). qRT-PCR was performed using SYBR *Premix Ex Taq* II (Takara Biomedical Technology Co., Ltd) with a LightCycle System (Roche, USA). The mRNA level of the *SOD2* gene was estimated by normalizing with β-actin. Sequences of the specific PCR primers for MnSOD: forward primer, 5’-AGCATGTTGAGCCGGGCAGT-3′; and reverse primer, 5’-AGGTTGTTCACGTAGGCCGC-3′; for β-actin: forward primer, 5’-CCTCAATTGATTCACCCACC-3′; and reverse primer, 5’-GCTGCTCTCCCCAAGGAT-3′.

### Chromatin immuneprecipitation (ChIP)

A ChIP-IT system (Active Motif, USA) was used to quantify RelB binding to the enhancer region of the *SOD2* gene according to the manufacturer’s protocol. Chromatin isolated from the treated cells was pulled down using a RelB antibody (#10544, Cell Signaling Technology). Unprecipitated chromatin was used as an input control and chromatin pulled down by an IgG antibody (Santa Cruze Biotechnology) served as a negative antibody control. The pulled down enhancer fragment was quantified using a quantitative PCR with gene specific primers: forward, 5’-CGGGGTTATGAAATTTGTTGAGTA-3′; and reverse, 5’-CCACAAGTAAAGGACTGAAATTAA-3′. Amounts of the pulled down fragment were assessed by normalizing with the input control.

### Animal experiment

Animal experiments were performed according to the Institutional Animal Care and Use approved by the Research Committee of Nanjing Medical University (No. IACUC-1601229). Five-week-old male nude (BALB/c) mice (Beijing Vital River Laboratory Animal Technology Co., Ltd., China) were used for mouse xenograft tumor experiments. 5 × 10^6^ PC-3 cells were subcutaneously implanted into the right flanks of mice. After tumor volume reaching to 500 mm^3^, the mice were randomly divided into four groups (10 mice in each group): saline control, 4 mg/kg of HZ08, 15 Gy IR and combined HZ08 and IR. HZ08 was injected through tail vein 1 h before IR treatment which was given every other day for 5 × 3 Gy. Tumor volume was measured using digital calipers every other day and calculated using a standard formula (V = 0.52 × AB^2^, A and B represent the diagonal tumor lengths). The mice were executed when tumor volume reached to 2000 mm^3^ and tumor tissues were removed for the following experiments.

### Statistical analysis

Data were presented as the mean ± standard deviation (SD) from at least three replicates. Significant differences between the experimental groups were analyzed by unpaired Student’s t-test. One-way analysis of variance (ANOVA) followed by Dunnett’s or Bonferroni’s multiple comparison test was performed using Prism (GraphPad, San Diego, USA). Statistical significance was accepted at *P* < 0.05.

## Results

### Downregulation of RelB enhances the radiosensitivity of PC-3 cells

**Fig. 1:**
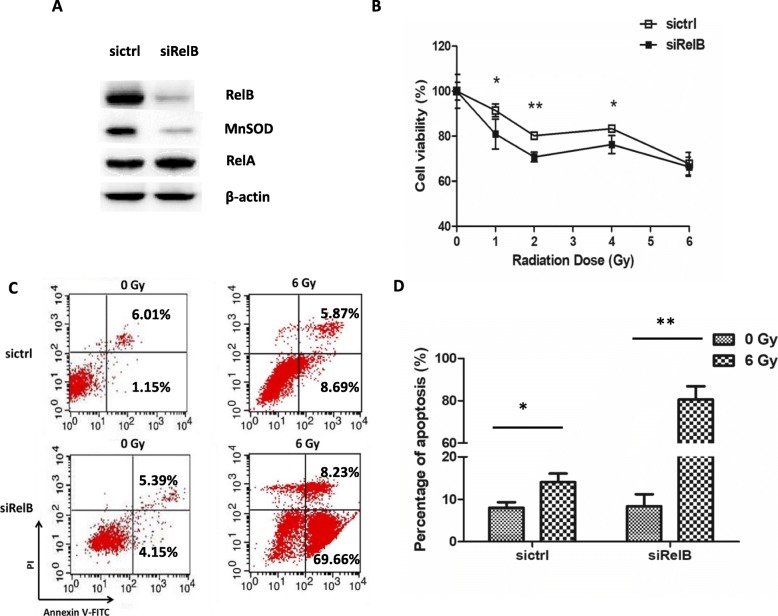
Radiosensitization of PC-3 cells by down-regulation of RelB. **a**, the endogenous RelB in PC-3 cells was silenced by stably transfecting a RelB siRNA construct. The levels of RelB and its relating proteins were quantified by Western blots. **b**, the cells were treated with a serial dosage of IR as indicated and cytotoxicity was analyzed by MTT assay. **c** and **d**, after IR treatment, proapoptotic and apoptotic cells were detected by flow cytometry (**c**), and percentage of apoptotic cells was calculated and plotted (**d**). Mean ± SD was representative of three independent experiments carried out in duplication. *(*P* < 0.05), **(*P* < 0.01) show the significances between two groups as indicated

Our previous studies reported that the high levels of nuclear RelB in PCa contribute to tumor metastatic progression and resistance to radiation [26, 28]. To investigate the role of RelB in radioresistance of PC-3 cells, we have previously established a RelB silenced PC-3 cells. As shown in Fig. 1a, after RelB was knocked down in PC-3 cells, NF-κB-regulated MnSOD protein was sharply reduced accordingly. Since the expression of RelA was slightly increased in the RelB-silenced PC-3 cells, the reduction of MnSOD was mainly caused by silencing RelB. To test whether the downregulation of RelB enhances the radiosensitivity, the RelB-silenced PC-3 cells were treated with different doses of IR and cytotoxicity in the cells was analyzed by MTT assay. Compared to PC-3 parent cells, the cell death was significantly increased in RelB-silenced cells after treated with radiation (Fig. 1b). In addition, the results from flow cytometry analysis showed that although knockdown of RelB slightly induced apoptosis, IR significantly induced apoptosis in the RelB-silenced cells (Fig. 1c and d). These results verify that RelB is a main contributor for radioresistance of PC-3 cells and suggest a promising approach for enhancing radiotherapeutic efficiency by targeting RelB.

### HZ08 enhances the radiosensitivity of PCa cells

**Fig. 2:**
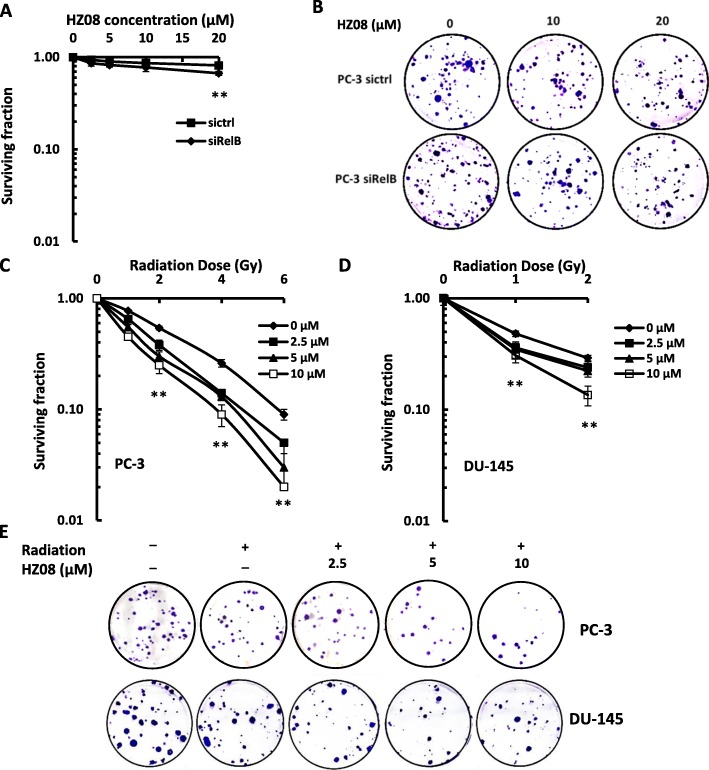
The effect of HZ08 on radiosensitization of PCa cells. **a** and **b**, PC-3 and RelB-silenced PC-3 cells were treated with a serial concentration of HZ08 as indicated and cell survival was analyzed by colony formation. **c**-**e**, PC-3, DU-145 cells were pretreated with HZ08 and followed by IR treatment as indicated. Colony survival assay was performed to quantify cell death. Colony surviving fractions were calculated from three independent experiments and plotted as mean ± SD. **(*P* < 0.01) shows the significances between two groups as indicated

HZ08 (Additional file 1: Figure S1), has been shown to be able to reverse drug resistance in a series of multidrug resistant cancers partially through inhibition of P-glycoprotein mediated drug efflux [22, 24, 29, 30]. To determine the cytotoxic effect of HZ08 in PCa cells, PC-3 and its RelB-silenced cells were treated with a serial concentration of HZ08 and cytotoxicity was quantified by both MTT (Additional file 2: Figure S2) and colony survival assays (Fig. 2a-b). HZ08 slightly induced cytotoxicity in PC-3 cells, but the effect was enhanced in RelB-silenced PC-3 cells. To examine whether HZ08 affects radioresistance of aggressive PCa cells, PC-3 and DU-145 cells were pretreated with HZ08 and followed by multiple doses of IR treatment. Because DU-145 cells appeared to be more sensitive to IR than PC-3 cells, the combined treatment were conducted by using low doses of IR to treat DU-145 cells. Although HZ08 alone appeared to have certain cytotoxic effect, HZ08 combined with IR significantly enhanced the radiosensitivity of PCa cells. Importantly, the synergetic effects on cell killing were obtained in the experimental groups by combination of low concentrations of HZ08 with low doses of IR (Fig. 2c-e). Since HZ08 was efficient to enhance IR-induced cell death by sufficiently suppressing RelB in PCa cells, the RelB-silenced PC-3 cells were not included in the following experiments for further investigating the relating mechanism.

### HZ08 increases IR-induced ROS production

**Fig. 3:**
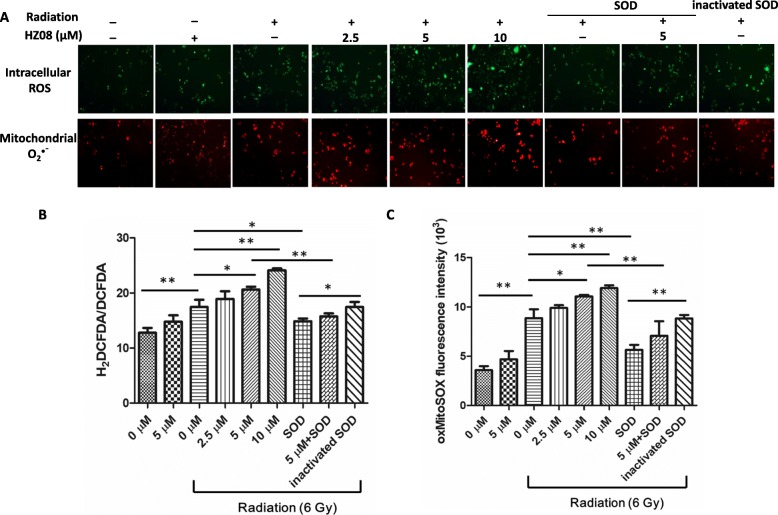
The effect of HZ08 on ROS production. **a**, PC-3 cells were treated with HZ08 and IR as indicated. ROS and superoxide anion were quantified using H_2_DCF-DA (green dye) and MitoSOX (red dye) reagents, respectively. **b**, DCFDA was used to normalize H_2_DCF-DA images. **c**, MitoSOX fluorescence intensity was quantified. To remove the treatment-induced oxidative free radicals, SOD or its heat-inactivated control was added into the cells. Mean ± SD was representative of three independent experiments carried out in duplication. *(*P* < 0.05), **(*P* < 0.01) show the significances between two groups as indicated

Induction of ROS generation is a major cause for radiation-mediated cell death. Thus, we speculated that HZ08-mediated radiosensitization effect is presumably by further elevating the levels of ROS. After HZ08 and IR treatments, the intracellular levels of ROS in PC-3 cells were quantified using H_2_DCF-DA reagent. As expected, the levels of green fluorescence-labeled total cellular ROS were highly increased in HZ08 and IR combined treatment group compared to IR alone treated group. However, pretreating with SOD was able to significantly attenuate cellular ROS caused by IR alone or IR combined with HZ08 (Fig. 3a and b). Meanwhile, mitochondrion-derived O_2_^•ˉ^ production was evaluated using MitoSOX reagent by assessing mitochondrial accumulation of superoxide based on its hydrophobic nature and positively charged triphenylphosphonium moiety. MitoSox oxidation was higher in IR and HZ08 combined treatment group compared with IR alone group. Consistently, MitoSox oxidation was decreased when the cells were pretreated with SOD (Fig. 3a and c).

### HZ08 decreases IR-induced mitochondrial respiration

**Fig. 4:**
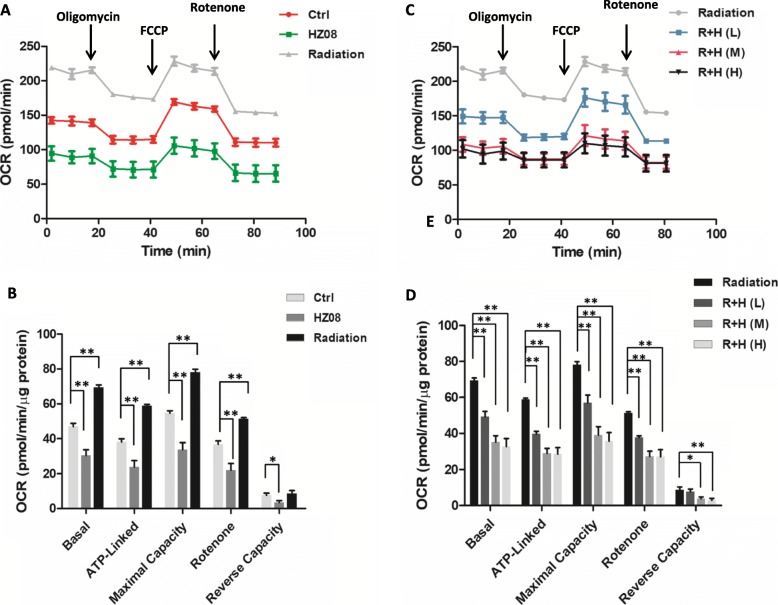
The effect of HZ08 on mitochondrial respiration. **a** and **b**, PC-3 cells were treated with 5 μM HZ08 or 6 Gy IR. Mitochondrial OCR in the cells was analyzed using a Seahorse XF-96 analyzer. Supplemental reagents including oligomycin, FCCP, rotenone were automatically injected into the analyzing plates to determine basal, ATP-linked and maximal OCR as well as background image, respectively. The OCR reverse capacity, an important mitochondrial respiration index, was calculated by the maximal OCR subtracted the basal OCR. **c** and **d**, PC-3 cells were pretreated with HZ08 at different doses as 2.5 μM (L), 5 μM (M) and 10 μM (H), and then followed by 6 Gy IR. Mitochondrial OCR in the treated cells was quantified accordingly. Mean ± SD was representative of three independent experiments carried out in duplication. *(*P* < 0.05), **(*P* < 0.01) show the significances between two groups as indicated

In addition, to determine whether the combined treatment affects the mitochondrial respiration rate in the cells, mitochondrial OCR was quantified using a Seahorse Metabolic Analyzer. HZ08 and IR exhibited the opposed effects on mitochondrial respiration. Mitochondrial OCR in PC-3 cells was increased by IR, but it was decreased by HZ08 (Fig. 4a and b). Pretreating with HZ08 not only efficiently abrogated IR-mediated OCR induction but also decreased the OCR levels even lower than the untreated control. Importantly, the suppressive effect of HZ08 on IR-enhanced mitochondrial respiration was reflected in a HZ08 dose-dependent manner (Fig. 4c and d).

### HZ08 represses MnSOD transcription via inactivation of RelB

**Fig. 5:**
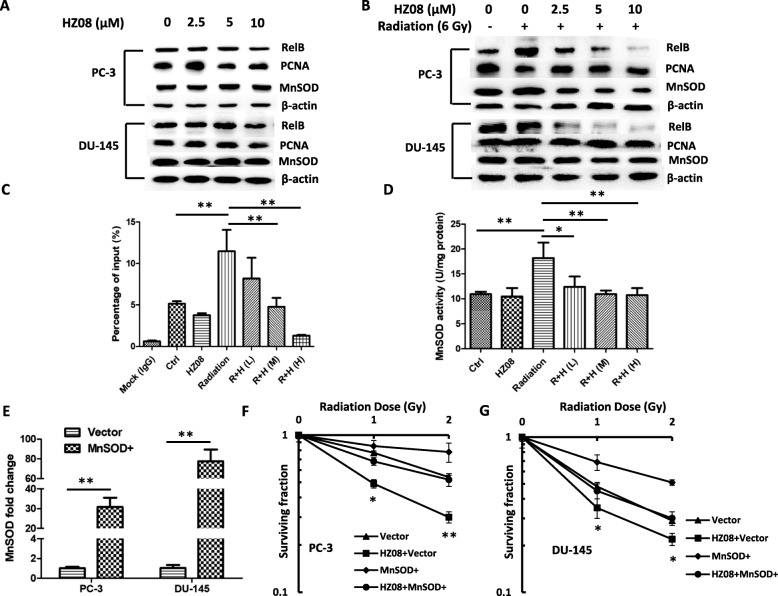
The effect of HZ08 on IR-induced RelB nuclear translocation and MnSOD expression. **a**, PC-3 and DU-145 cells were treated with different concentrations of HZ08. The levels of nuclear RelB and relating cytosolic MnSOD were quantified by Western blots. PCNA and β-actin served as the loading controls. **b**, the two cell lines were pretreated with HZ08 and then followed by IR treatment as indicated. The amounts of RelB and MnSOD in the fractions of nuclei and cytosols were quantified accordingly. **c**, PC-3 cells were treated with HZ08 and IR as described in Fig. 4c. Chromatin was isolated from the treated cells and pulled down using a RelB antibody or IgG control. An enhancer fragment of the *SOD2* gene containing a NF-κB element was amplified by qPCR with specific primers. The amount of pulled down fragment was quantified by normalizing amplified from unprecipitated chromatin (input control). **d**, after treatment, the cell extracts were subjected to measure MnSOD activity. **e**-**g**, PC-3 and DU-145 cells were transfected with a MnSOD expression construct, and then treated with HZ08 and IR. The increased level of MnSOD mRNA was confirmed by qRT-PCR with β-actin normalization (**e**). Cell survival was quantified by colony formation (**f** and **g**). Mean ± SD was representative of three independent experiments carried out in duplication. *(*P* < 0.05), **(*P* < 0.01) show the significances between two groups as indicated

Our previous studies demonstrated that IR-generated ROS adaptively induces MnSOD expression in PCa cells mainly through activation of RelB-based NF-κB alternative pathway, which is considered to subsequently increase mitochondrial respiration [31]. The above results demonstrated that HZ08 suppresses mitochondrial respiration leading to the enhanced radiotherapeutic efficiency in PCa cells (Figs. 2, 3 and 4). Thus, it has been speculated that the radiosensitization effect of HZ08 may be mediated through down-regulation of MnSOD by suppressing RelB. To determine the effect of HZ08 on RelB nuclear translocation, nuclei and cytosols were separated to analyze the levels of nuclear RelB and MnSOD. In contrast to IR, HZ08 treatment seemed to change neither nuclear RelB nor cytoplasmic MnSOD (Fig. 5a). Intriguingly, pretreating with HZ08 dramatically attenuated IR-induced RelB and MnSOD in dose-dependent manners, suggesting that HZ08 is able to prevent RelB nuclear translocation and down-regulate MnSOD in the irradiated cells (Fig. 5b).

Furthermore, to mechanistically confirm that RelB transcriptional regulation of MnSOD is a main mechanism for radiosensitization effect of HZ08, the NF-κB element located in the enhancer region of the *SOD2* gene was pulled-down by a RelB antibody and the relating DNA fragment was further quantified by a quantitative PCR with gene specific primers. Consistently, 一iIR increased the precipitated enhancer region, which was further eliminated by HZ08 (Fig. 5c). Accordingly, IR adaptively induced the MnSOD activity, but the IR effect was further removed by HZ08 (Fig. 5d). Finally, to verify whether MnSOD plays a key role in radioresistance of PCa cells, MnSOD was ectopically expressed in PC-3 and DU-145 cells (Fig. 5e). As anticipated, the overexpression of MnSOD could decrease IR-induced cytotoxicity, particularly the increase of MnSOD partially attenuated HZ08-mediated radiosensitization (Fig. 5f and g; Additional file 3: Figure S3A, B).

### HZ08 inhibits PI3K/Akt/IKKα phosphorylation in PCa cells

**Fig. 6:**
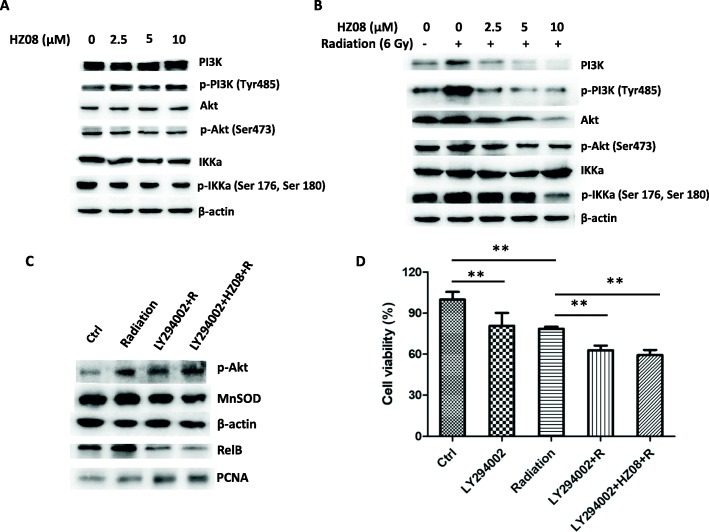
The effect of HZ08 on phosphorylation of PI3K, Akt and IKKα. **a** and **b**, PC-3 cells were treated with HZ08 alone (**a**) or treated with HZ08 and IR (**b**). Cellular extracts from the treated cells were used to quantify the levels of PI3K, p-PI3K, Akt, p-Akt, IKKα, p-IKKα by Western blots. **c** and **d**, PC-3 cells were pretreated with LY294002 (25 μM), HZ08 (5 μM) and treated with 6 Gy IR. P-Akt, MnSOD in cellular extracts and RelB in nuclear extracts were quantified by Western blots (**c**). Cytotoxicity was analyzed using MTT by three independent experiments and plotted as mean ± SD. ** (*P* < 0.01) shows the significances between two groups as indicated (**d**)

To elucidate the precise mechanisms by which HZ08 sensitizes PCa cells to radiation, we examined the upstream signaling involved in the activation of the NF-κB alternative pathway. IKKα, a member of the IκB kinase family, has been found to be a key factor in the activation of both the NF-κB classic and alternative pathways, especially the formation of its homodimer has been shown to be essential for the activation of alternative pathway [32]. Thus, we assessed the effect of HZ08 on IKKα phosphorylation in PC-3 cells. IKKα and its phosphorylated forms (p- IKKα at Ser 176 and Ser 180) were quantified by Western blots with a specific antibody. HZ08 seemed to be negative for IKKα phosphorylation (Fig. 6a). Consistent with the result of RelB nuclear translocation, IR alone significantly increased p-IKKα levels compared to the untreated control. However, pretreating with HZ08 efficiently abrogated IR-induced IKKα phosphorylation (Fig. 6b).

Furthermore, PI3K/Akt signaling was further examined for determination of the upstream signaling to stimulate IKKα phosphorylation. HZ08 slightly increased PI3K phosphorylation (Tyr485) but no observed effect on Akt phosphorylation (Ser473) (Fig. 6a). Importantly, IR highly induced both PI3K and Akt phosphorylation, which were completely eliminated by pretreating with HZ08 (Fig. 6b). To verify that PI3K/Akt/IKKα signaling axis is mainly responsible for HZ08-mediated radiosensitization in PCa cells, an inhibitor of PI3K (LY294002) was applied to validate its effect on prevention of RelB nuclear translocation. Expectedly, the levels of p-Akt, nuclear RelB and MnSOD were obviously reduced in LY294002-treated PC-3 cells (Fig. 6c). The cell survival rate was also significantly decreased accordingly when IR was combined with LY294002 (Fig. 6d). Nevertheless, there was no more radiosensitization effect observed when HZ08 was combined with LY294002, suggesting that the effect of HZ08 is mainly dependent on suppression of PI3K signaling. Altogether, these results suggest that PI3K/Akt/IKKα signaling axis plays a pivotal role in HZ08-mediated radiosensitization of PCa cells.

### Radiosensitization effect of HZ08 is validated in nude mice

**Fig. 7:**
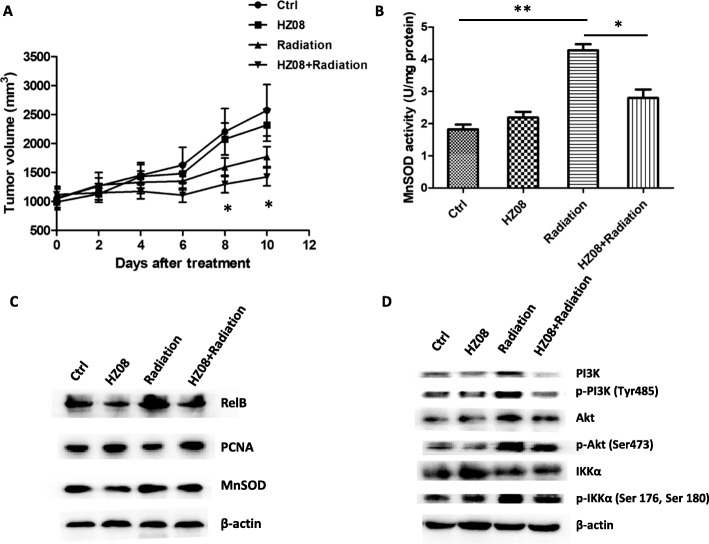
Validation of HZ08 effect on radiosensitization of PC-3 cells in vivo. **a**, 5 × 10^6^ PC-3 cells were subcutaneously injected into male nude mice to form tumors. The volumes of PC-3 bearing tumors were measured every other day. The mice were treated with HZ08 (4 mg/kg) and 5 × 3 Gy IR when the tumor volumes reached to 500 mm^3^. Tumor growth was observed daily until the volumes reached to 2000 mm^3^. **b**-**d**, Tumor tissues were homogenized and total proteins and nuclear proteins were extracted. The extracted total proteins were used to measure MnSOD activity (**b**) and quantify amounts of MnSOD and relating signal proteins at the upstream (**c** and **d**). The extracted nuclear proteins were used to quantify the level RelB in nuclei (**c**). *(*P* < 0.05), **(*P* < 0.01) show the significances between two groups as indicated

A mouse xenograft tumor model was used to validate the radiosensitization effect of HZ08 in PC-3 cells. Male nude mice were subcutaneously injected with PC-3 cells and allowed to form tumors. When tumor volumes reached to 500 mm^3^, the mice were divided into four groups for the treatment as described above. In the control group, the tumors rapidly grew to reach the maximum volume (2000 mm^3^) in only 10 days. HZ08 alone seemed to have no toxic effect on the nude mice but slightly reduced the tumor growth rate. IR was essential to prevent the tumor growth and its therapeutic capacity was further enhanced when combined with HZ08 (Fig. 7a, Additional file 4: Figure S4). Ten days after treatment, the mice were humanely killed and the tumor tissues were excised. Total proteins and nuclear proteins were extracted from the tumor tissues and subjected to measure MnSOD activity and analyze the levels of the relating proteins. IR highly induced MnSOD activity, but it was removed by HZ08 (Fig. 7b). Accordingly, the nuclear levels of RelB and cellular MnSOD were also increased by IR and further eliminated by HZ08 (Fig. 7c). Subsequently, although IR elevated the expression levels of PI3K, Akt and IKKα as well as amounts of their phosphorylated forms, HZ08 also efficiently impeded the IR effects on induction of PI3K/Akt/IKKα phosphorylation (Fig. 7d). Combined to the results from in vitro experiments, the data suggest that HZ08-enhanced radiosensitivity of PCa cells is, at least in part, through repression of MnSOD adaptive transcriptional activation by inhibiting IR-induced PI3K/Akt/IKKα signaling pathway.

## Discussion

PCa remains a large health burden in the advanced countries and its morbidity also rapidly increases in the developing countries such as China. In the United States, PCa is the most common cancer in men, about 1 in 7 men will be diagnosed with PCa during their lifetime [33]. Since radiotherapy is thought to be a main option to treat localized PCa, the maximal tolerated radiation doses have already been tested to treat advanced PCa. However, keeping increasing radiation doses may not contribute to clinical benefit because the high doses of radiation imperatively cause radiotoxicity to normal tissues. Thus, it is urgently needed to discover novel radiosensitizers for improving radiotherapeutic efficacy, particularly sensitizing PCa cells to low doses of radiation. Since increasing ROS production is a major contributor for HZ08-mediated anticancer therapeutic enhancement, we speculated that HZ08 may be an ideal radiosensitizing agent that can efficiently regulate cellular redox response under radiotherapeutic conditions.

Radioresistance of cancer cells is mainly mediated by increasing capacity of antioxidant defense in response to high levels of ROS. Thus, it is a promising approach to enhance radiotherapeutic efficacy by suppression of cellular antioxidants. We and others have demonstrated that down-regulation of MnSOD is essential for sensitizing PCa cells to radiation. The present study further delineated that HZ08 increases IR-inducible ROS generation due to suppression of MnSOD. In general, up-regulation of MnSOD leads to increasing mitochondrial respiration due to more efficient removal of ROS, resulting in acceleration of mitochondrial respiration for cell survival and proliferation. The present study provided a proof-of-concept evidence that IR-induced MnSOD expression led to increasing mitochondrial OCR and further invalided by HZ08, suggesting that the effect of HZ08 on radiosensitization of PCa cells is ascribed to abrogation of cell protective function mediated by MnSOD.

The activation of NF-κB signaling pathway has been ascertained to contribute toward tumor resistance to radiotherapy and chemotherapy [34–36]. Thus, inhibition of NF-κB has been contemplating as a useful approach to enhance the efficacy of conventional radiotherapy and chemotherapy [37]. NF-κB can be triggered by both classical and alternative pathways, which further regulates different sets of target genes involved in immune and inflammatory responses based on the nuclear translocation of p50/RelA and p52/RelB dimers, respectively [38]. Previously, we have reported that RelB highly expresses in aggressive PCa cells’ [31]. Correspondently, the suppression of RelB is beneficial for radiation to treat advanced PCa [39]. The present study further demonstrated that the radiosensitivity of PCa cells is enhanced by down-regulating RelB through either RelB siRNA transfection or HZ08 treatment. NF-κB, a redox sensitive transcription factor, appears to be inducible in response to ROS stimulated by a variety of inflammatory agents. As a consequence of the NF-κB activation, up-regulation of MnSOD expression is necessary for protecting the cells against massive ROS. Conversely, HZ08 suppresses MnSOD by inactivating RelB, leading to promotion of IR-mediated cell death by increasing ROS. Taken together, these results further verified that inhibition of NF-κB signaling pathway is a promising approach for enhancing radiotherapeutic efficiency to control advanced PCa.

**Fig. 8:**
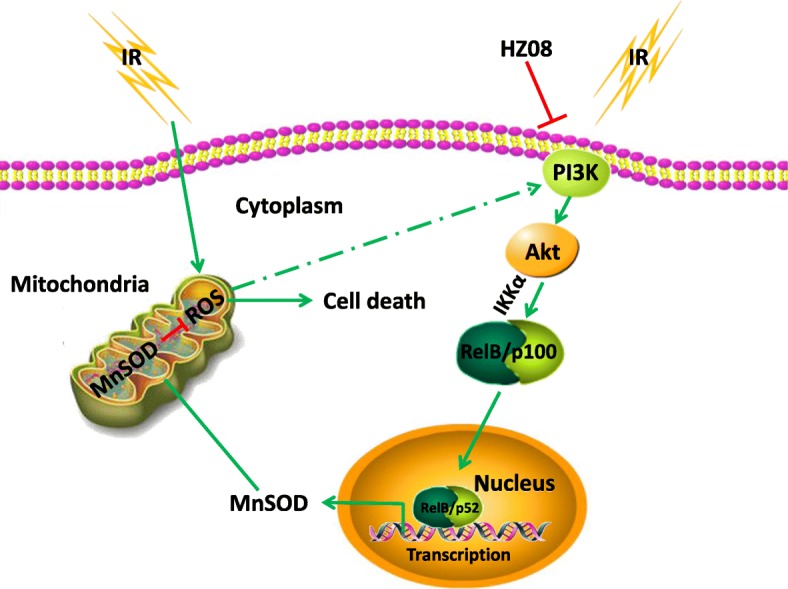
Depiction of suggested mechanism involved in HZ08-mediated radiosensitization of PC-3 cells

NF-κB is constitutively expressed at high levels in many types of cancer and it can be activated through multiple signaling pathways in response to radiotherapy and chemotherapy [13, 40, 41]. In the classic pathway, the formation of IKK complex by IKKα-IKKβ dimer linking to IKKγ is sufficient for phosphorylation and degradation of IκB, the main inhibitor of RelA/p50 nuclear translocation. Whereas the formation of IKKα-IKKα homodimer led to processing of p100 to p52 and promoting of p52/RelB nuclear translocation has been defined in the alternative pathway [42, 43]. Thus, it is recognized that IKKα activation plays a key role in activation of the alternative pathway. Although NIK was initially identified to be a main upstream kinase to phosphorylate IKKα [32], PI3K/Akt can also activate IKKα-dependent NF-κB alternative pathway in androgen-independent PCa cells [41]. The present study revealed that HZ08 enhances the radiosensitivity of PCa cells through suppression of the NF-κB alternative pathway by inhibiting PI3K/Akt/IKKα phosphorylation as illustrated in Fig. 8. Therefore, the use of therapeutic agents such as HZ08 may efficiently improve radiotherapy for treating advanced PCa with high constitutive levels of RelB.

Notably, the synergistic effect of HZ08 and IR on the treatment of PCa cells was validated using an applicable mouse xenograft tumor model. Body weight loss was observed in the period of tumor rapid growing but in turn gained after treating tumors with IR alone or combined with HZ08. Importantly, no detectible body weight loss was found when HZ08 was applied, indicating that HZ08 had no considerable toxic effect, by itself, on body weight and survival of mice implanted with PC-3 cells. Accordingly, HZ08 alone did not largely change the levels of RelB and MnSOD as well as phosphorylation levels of PI3K, Akt and IKKα, suggesting that HZ08 may not have meaningful toxic side effects on normal tissues.

Although radiotherapy is an effective option to treat localized PCa, its therapeutic efficacy eventually decreases when PCa develops radioresistance. Since adaptive response of antioxidant defense is a major cause for increasing radioresistance of PCa, it is imperative that the combination of antioxidant inhibitors with traditional radiotherapy may be beneficial to improve PCa treatment. The activation of cell signaling pathways including NF-κB and its upstream regulators is supposed to be a vital obstacle in the control of PCa. It is widely recognized therefore, the discovery of applicable adjuvant drugs for blockage of such signaling pathways is necessary for improving the efficiencies of radiotherapy and chemotherapy. The finding of HZ08 anticancer capacity suggests that HZ08 may be a novel drug candidate to enhance the conventional radiotherapy and chemotherapy. Indubitably, for clinical use of HZ08 as a potential radiosensitizer in the treatment of advanced PCa, there should be more detail pharmacological studies needed to be conducted, particularly to decide clinical applicable treatment strategy for enhancing the therapeutic efficiency using low doses of radiation.

## Conclusions

Although radiotherapy is an effective option to treat localized PCa, its therapeutic efficacy decreases when PCa develops radioresistance. RelB is highly expressed in advanced PCa patients suggesting that activation of RelB-based NF-κB alternative pathway is critical for PCa progression. Here, we report that HZ08 sensitizes aggressive PCa cells to IR treatment through suppression of RelB nuclear translocation. Mechanistically, HZ08 inhibits PI3K/Akt/IKKα signaling axis, leading to repression of RelB-mediated transcriptional of MnSOD.

## Additional files

Additional file 1 : Figure S1. HZ08 chemical structure. (PDF 211 kb)

Additional file 2 : Figure S2. The cytotoxic effect of HZ08 in PC-3 cells. PC-3 and RelB-silenced PC-3 cells were treated with a serial concentration of HZ08 as indicated and cytotoxicity was analyzed by MTT assay. Mean ± SD was representative of three independent experiments carried out in duplication. **(*P* < 0.01) shows the significances between two groups as indicated. (PDF 206 kb)

Additional file 3 : Figure S3. The reverse effect of transfected MnSOD on cell viability of HZ08 and IR-treated cells. PC-3 (A) and DU-145 cells (B) were transfected with a MnSOD expression construct, and then treated with 5 μM HZ08 and 6 Gy IR. Cell viability was quantified by MTT. Mean ± SD was representative of three independent experiments carried out in duplication. **(P < 0.01) shows the significances between two groups as indicated. (PDF 390 kb)

Additional file 4 : Figure S4. The effect of HZ08 on radiosensitization of PC-3 cell bearing tumors in nude mice. The tumor volumes of mice with different treatments were indicated above. (PDF 228 kb)

## Abbreviations

ChIP: Chromatin immuneprecipitation
DCF: Dichlorofluorescein
HZ08: 6,7-dimethoxy-1-(3,4-dimethoxy)benzyl-2-(N-n-octyl-N′-cyano) guanyl-1,2,3,4-tetrahydroisoquinoline
IR: Ionizing radiation
MnSOD: Manganese-dependent superoxide dismutase
NF-κB: Nuclear factor-κb
OCR: Oxygen consumption rates
PCa: Prostate cancer
qRT-PCR: Quantitative reverse transcription polymerase chain reaction
ROS: Reactive oxygen species
siRNA: Small interfering RNA
