# Supplementary figures and images for: HZ08 suppresses RelB-activated MnSOD expression and enhances Radiosensitivity of prostate Cancer cells
Source: J Exp Clin Cancer Res. 2018 Jul 27;37:174. doi: 10.1186/s13046-018-0849-5 (PMC6062957; doi:10.1186/s13046-018-0849-5)

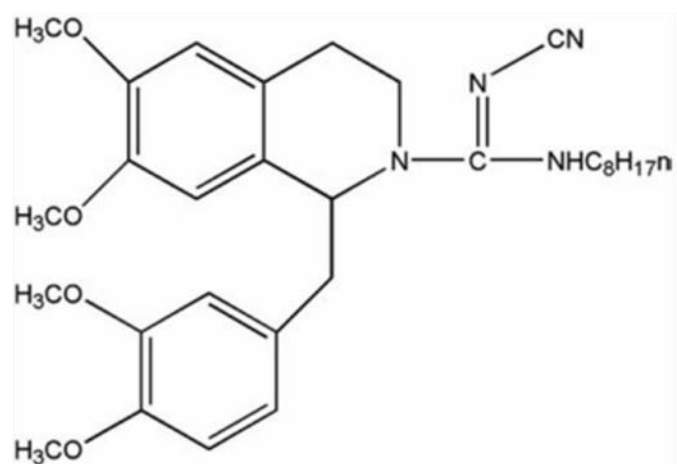

**Fig. S1. HZ08 chemical structure.**

Supplement: Supplementary file 1 — : Figure S1. HZ08 chemical structure. (PDF 211 kb) [file 13046_2018_849_MOESM1_ESM.pdf]
