# Supplementary material for: HZ08 suppresses RelB-activated MnSOD expression and enhances Radiosensitivity of prostate Cancer cells
Source: J Exp Clin Cancer Res. 2018 Jul 27;37:174. doi: 10.1186/s13046-018-0849-5 (PMC6062957; doi:10.1186/s13046-018-0849-5)
Supplement: Supplementary file 2 — : Figure S2. The cytotoxic effect of HZ08 in PC-3 cells. PC-3 and RelB-silenced PC-3 cells were treated with a serial concentration of HZ08 as indicated and cytotoxicity was analyzed by MTT assay. Mean ± SD was representative of three independent experiments carried out in duplication. **(P < 0.01) shows the significances between two groups as indicated. (PDF 206 kb) [file 13046_2018_849_MOESM2_ESM.pdf]

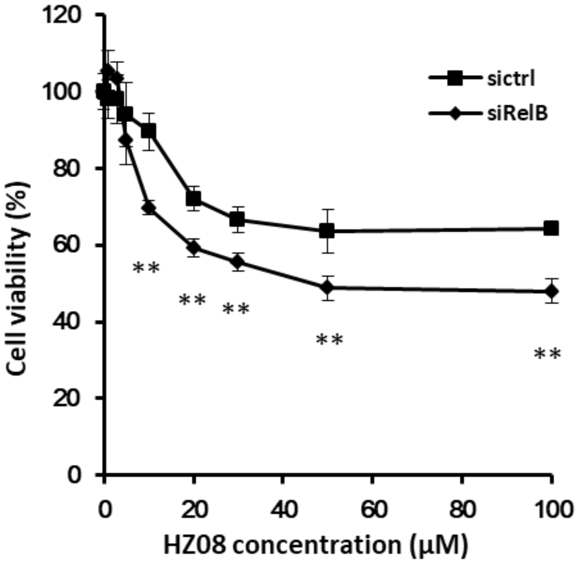

Fig. S2. The cytotoxic effect of HZ08 in PC-3 cells. PC-3 and RelB-silenced PC-3 cells were treated with a serial concentration of HZ08 as indicated and cytotoxicity was analyzed by MTT assay. Mean ± SD was representative of three independent experiments carried out in duplication. **(*P*< 0.01) shows the significances between two groups as indicated.
