# Supplementary material for: HZ08 suppresses RelB-activated MnSOD expression and enhances Radiosensitivity of prostate Cancer cells
Source: J Exp Clin Cancer Res. 2018 Jul 27;37:174. doi: 10.1186/s13046-018-0849-5 (PMC6062957; doi:10.1186/s13046-018-0849-5)
Supplement: Supplementary file 3 — : Figure S3. The reverse effect of transfected MnSOD on cell viability of HZ08 and IR-treated cells. PC-3 (A) and DU-145 cells (B) were transfected with a MnSOD expression construct, and then treated with 5 μM HZ08 and 6 Gy IR. Cell viability was quantified by MTT. Mean ± SD was representative of three independent experiments carried out in duplication. **(P < 0.01) shows the significances between two groups as indicated. (PDF 390 kb) [file 13046_2018_849_MOESM3_ESM.pdf]

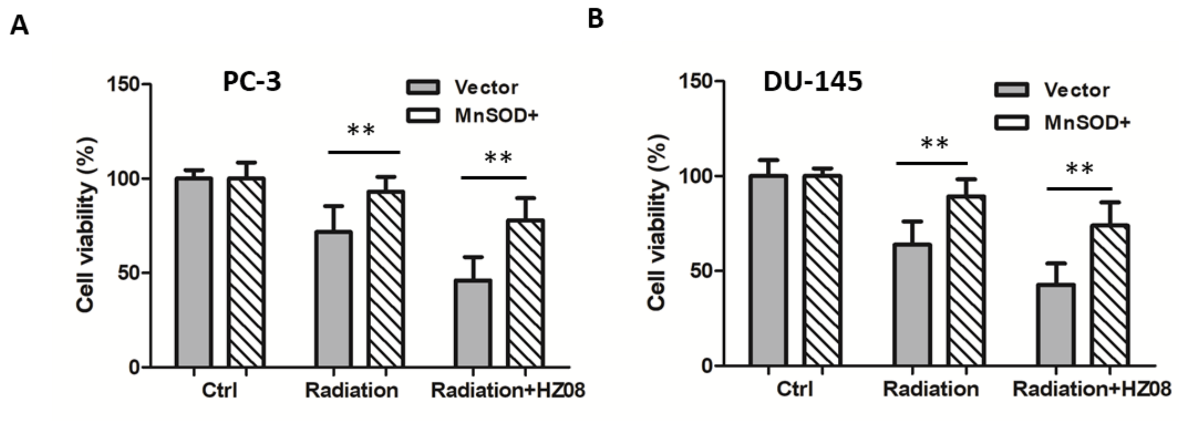

Fig. S3. The reverse effect of transfected MnSOD on cell viability of HZ08 and IR-treated cells. PC-3 (A) and DU-145 cells (B) were transfected with a MnSOD expression construct, and then treated with 5 μM HZ08 and 6 Gy IR. Cell viability was quantified by MTT. Mean ± SD was representative of three independent experiments carried out in duplication. **(*P*< 0.01) shows the significances between two groups as indicated.
