# Supplementary material for: HZ08 suppresses RelB-activated MnSOD expression and enhances Radiosensitivity of prostate Cancer cells
Source: J Exp Clin Cancer Res. 2018 Jul 27;37:174. doi: 10.1186/s13046-018-0849-5 (PMC6062957; doi:10.1186/s13046-018-0849-5)
Supplement: Supplementary file 4 — : Figure S4. The effect of HZ08 on radiosensitization of PC-3 cell bearing tumors in nude mice. The tumor volumes of mice with different treatments were indicated above. (PDF 228 kb) [file 13046_2018_849_MOESM4_ESM.pdf]

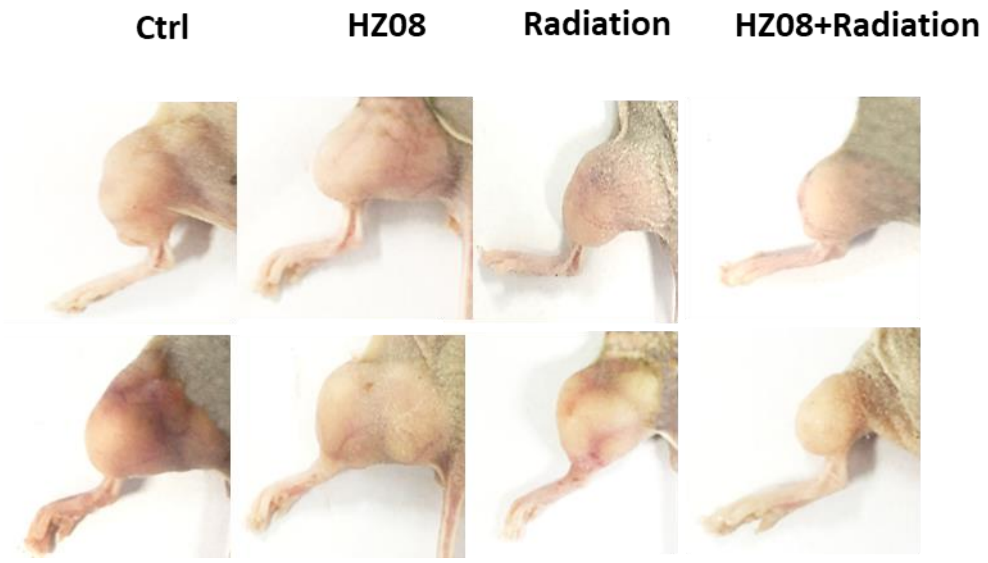

Fig. S4. The effect of HZ08 on radiosensitization of PC-3 cell bearing tumors in nude mice. The tumor volumes of mice with different treatments were indicated above.
